# Searching Before It Is Too Late: A Survey of Blood Parasites in *Ctenosaura melanosterna*, a Critically Endangered Reptile of Honduras

**DOI:** 10.5402/2013/495304

**Published:** 2012-11-28

**Authors:** Andrew K. Davis, Andrew C. Benz, Leslie E. Ruyle, Whitney M. Kistler, Barbara C. Shock, Michael J. Yabsley

**Affiliations:** ^1^Odum School of Ecology, The University of Georgia, Athens, GA 30602, USA; ^2^Division of Biological Sciences, The University of Georgia, Athens, GA 30602, USA; ^3^Applied Biodiversity Science Program, Texas A&M University, College Station, TX 77843-2258, USA; ^4^Warnell School of Forestry and Natural Resources, The University of Georgia, Athens, GA 30602, USA; ^5^The Southeastern Cooperative Wildlife Disease Study, Department of Population Health, College of Veterinary Medicine, The University of Georgia, Athens, GA 30602, USA

## Abstract

For species at risk of extinction, any parasites they have would be expected to face a similar fate. In such cases, time is running out for efforts to identify and study their parasitic fauna before they are gone. We surveyed the hemoparasite fauna of 50 black-chested, spiny-tailed iguanas (*Ctenosaura melanosterna*), a critically-endangered species, on an island off the coast of Honduras. Blood samples from captured animals were tested for hemoparasites by thin blood smear and molecular analyses. Based on microscopy, two parasites were identified, a *Plasmodium* sp. in 14% of iguanas and a *Hepatozoon* sp. in 32%. For both parasites, parasitemia levels were <0.1%. Prevalence and parasitemias of *Hepatozoon* declined with increasing host size, a pattern differing from most prior studies of saurian reptiles. From a subset of iguanas with microscopy-confirmed *Plasmodium* infections, sequence analysis of 454 bp of the cytochrome b gene indicated that the *Plasmodium* species was distinct from known *Plasmodium* and was most closely related to *P. chiricahuae* (96.5% similarity) followed by *P. mexicanum* (95.8% similarity). Efforts to amplify the *Hepatozoon* parasite using PCR were not successful. Additional surveys and studies of this host-parasite system would be valuable, both to science and to the management of this endangered animal.

## 1. Introduction

 When an entire species is in danger of becoming extinct, those species that depend on the endangered species, such as its parasite fauna, are also doomed to extinction unless the host species can be protected. In many cases, especially with endangered species that have been little studied, their associated parasites are being lost even before they can be observed, classified, and formally described [1]. In addition, hosts that are threatened often have limited ranges and/or small populations, which can further reduce their parasite fauna [2]. For many years the task of identifying the parasites associated with organisms of conservation concern was not a priority, or if it was, it was only because they were considered threats to conservation goals [3]. In more recent years, there has been a shift in thinking about the integral role of parasites in ecosystems, along with a new effort to impress upon conservationists, veterinarians, and laypersons that the preservation of biodiversity, one of the fundamental objectives of conservation, should include parasites [3–5]. At the very least, all efforts should be made to identify parasites in any hosts that are on a path to extinction, lest they disappear before being documented by science. 

**Figure 1:**
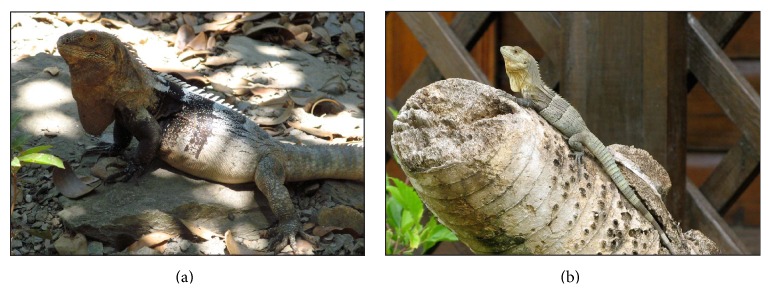
Photographs of adult (a) and juvenile (b) black-chested spiny-tailed iguanas (*C. melanosterna*). Photos taken by L. Ruyle on Cayo Menor, Honduras.

The black-chested, spiny-tailed iguana (*Ctenosaura melanosterna*, Figure 1) is a reptile species that typifies the above scenario. It is a medium to large-sized lizard that inhabits a small region of Honduras: the Rio Aguan Valley of the mainland and the nearby Cayos Cochinos archipelago [6]. Because of this limited geographic range, plus high levels of habitat loss and anthropogenic pressure, both the mainland and island populations have been listed as critically endangered by the IUCN Red List [7]. A recent genetic analysis also indicates that the island populations are distinct enough from mainland populations to be considered their own evolutionarily significant unit [7]. Little is known of the biology and natural history of mainland or island populations, and to our knowledge no studies have examined the blood parasite fauna of this species although some efforts have been made to examine other reptile species on Isla de Roatan, Honduras [8]. Here we report the results of a survey of blood parasites from wild-caught *C. melanosterna* (on an island population), and in which we discovered (using light microscopy and PCR) two distinct parasites, one of which appears to be a previously undescribed species of *Plasmodium*.

## 2. Materials and Methods

### 2.1. Study Site and Trapping Procedures

 All animals were captured on the island of Cayo Cochino Menor (Little Hog Island) in the Cayos Cochinos archipelago off the Atlantic coast of Honduras, as a part of a larger capture mark recapture (CMR) project to understand the population ecology of this critically endangered species [9]. Cayo Cochino Menor has an area of approximately 65 hectares and is 1.5 kilometers from north to south and 1.1 kilometers from east to west with a highest elevation point of 140 m. For additional details of the study site see Davis et al. [10]. Iguanas were captured with either baited wire mesh traps or by noose [10]. Upon capture we determined the gender of each iguana (if possible) and measured its snout-vent length (in cm), which served as an index of body size. We then collected 0.2 ml of blood from the caudal vein of each iguana using a 1.0 ml syringe and needle. With this sample we made a standard blood film for parasite assessment and stored the rest of the whole blood in Longmire's solution. All iguanas were released at the site of capture.

### 2.2. Examination of Blood Films

For the purposes of this project, we examined blood films from a total of 50 iguanas that were captured during July 2010. Slides were stained with a buffered Wright-Giemsa stain (Camco Quik Stain II) and examined with a light microscope under 1000X by one of us (ACB) for intracellular or extracellular blood parasites including hemogregarines, *Plasmodium*, *Trypanosoma*, and microfilaria [11]. At least 50 fields of view were scanned, and all parasites seen were recorded [12]. At this magnification, fields of view typically contain ~180–200 erythrocytes (AKD, *unpublished  data*). If no parasites were seen after 50 fields were examined, we classified the animal as uninfected.

### 2.3. Molecular Analyses

From a subset of iguanas that had confirmed infections via microscopy, we extracted DNA from 10 *μ*L of whole blood (preserved in Longmire's solution) using a Qiagen DNeasy blood and tissue kit per manufacturer's instructions (Qiagen, Valencia, CA, USA). Primers and a PCR protocol designed to amplify a portion of the cytochrome b gene of avian *Plasmodium* spp. and *Haemoproteus* spp. were used as described [13]. Amplicons were purified from agarose gels using a gel extraction kit (Qiagen) and submitted to the University of Georgia Genomics Facility for bidirectional Sanger sequencing. Our *Plasmodium* sequence was aligned with related sequences available in GenBank using MEGA v5.0, and phylogenetic relationships were analyzed using the neighbor-joining method with Kimura-2 parameter, a gamma distribution for mutation rates, and 1000 replicates to determine boot strap support [14]. Attempts to amplify the *Hepatozoon* observed in blood smears were made using primer pairs ITS-15C and ITS-13B, RLBH-F and RLBH-R, and 5.1 and B as described [15, 16].

## 3. Results

### 3.1. Light Microscopy Observations

**Figure 2:**
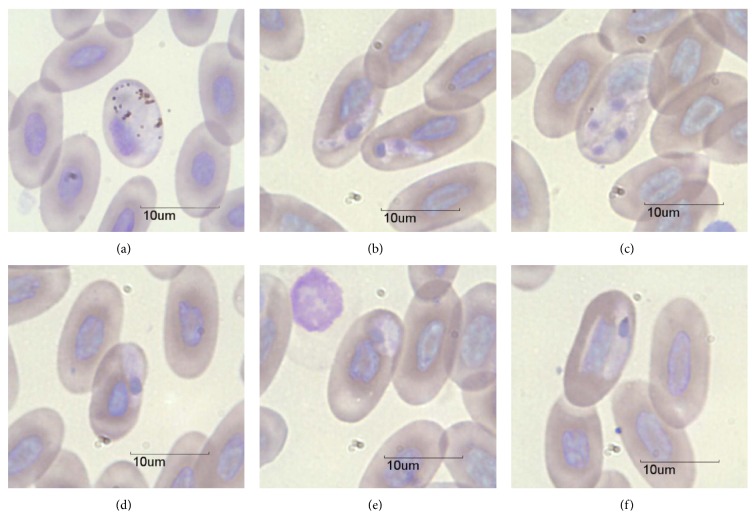
Photomicrographs of macrogametocytes of *Plasmodium* (a) and *Hepatozoon* (b)–(f) detected in *C. melanosterna *from Cayo Cochino Menor, Honduras.

 Two distinct parasite types were detected. The first was an intraerythrocytic organism morphologically consistent with *Plasmodium* macrogametocytes (confirmed via PCR, below; Figure 2(a)) that were detected in seven (14%) of the 50 iguanas. The macrogametocytes were typically round to oval shaped and contained numerous dark-staining granules, and the parasite encompassed most of the cell, often displacing the nucleus (Figure 2(a)). The average number of parasites seen in 50 fields was 2.2 (±2.13 SD). This translates to ~0.02% of cells (assuming ~200 cells per field). The second parasite we observed was morphologically consistent with that of a *Hepatozoon* ([11], Figures 2(b)–2(f)) and was detected in 16 (32%) iguanas. In most cases this intraerythrocytic organism was banana shaped with one to two (darkly-staining) nuclear regions and rarely displaced the host cell nucleus (except when two or more parasites were present in one cell, see Figure 2(c)). Occasionally we observed an oval-shaped variant (Figure 2(e)) that displayed similar staining characteristics. Based on measurements made of 40 random parasites (of the banana shaped type), this organism measured approximately 8.6 *μ*m × 2.2 *μ*m. The average number of *Hepatozoon* parasites seen in 50 fields was 6.1 (±15.5 SD), or approximately 0.06% of cells.

### 3.2. Infection Prevalence

**Figure 3:**
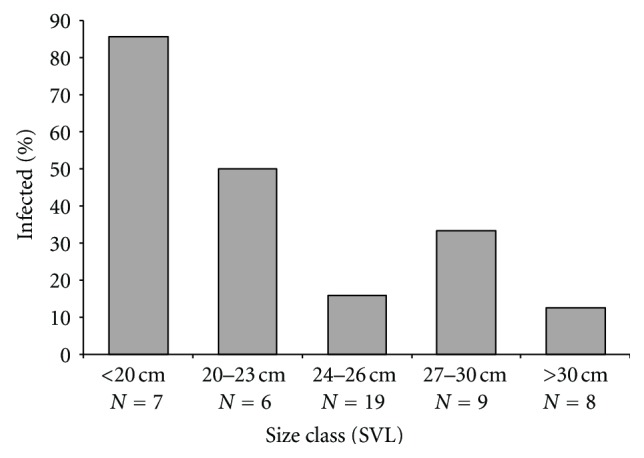
Prevalence of *Hepatozoon* infection based on iguana body size (snout-vent length).

**Figure 4:**
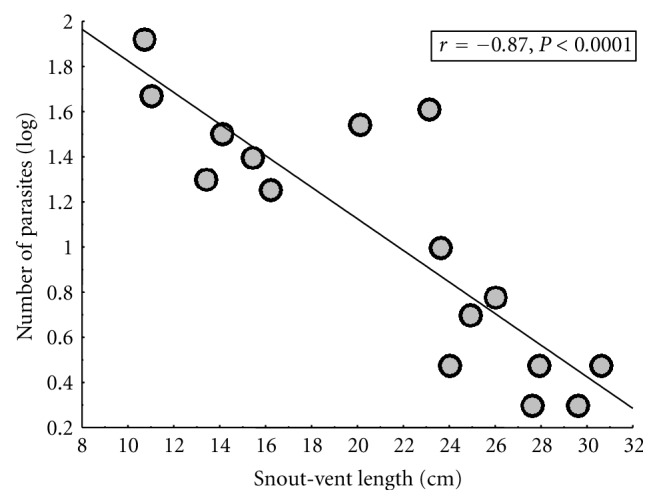
Relationship between *Hepatozoon* infection severity (number of parasites observed in 50 fields of view at 1000X, log transformed) and iguana body size.

The frequency of *Plasmodium* infections in male iguanas was higher than in females (20% versus 4.3%), though this difference was not significant (*χ* ^2^ = 2.683, *df* = 1, *P* = 0.101). The frequency of *Hepatozoon* infections in male and female iguanas was not different (*χ* ^2^ = 0.203, *df* = 1, *P* = 0.652); 8 out of 25 males were infected (32%) while 6 out of 23 females were infected (26%). Gender was not determined for two animals. Interestingly, there was a distinct relationship between *Hepatozoon* infection and body size; Figure 3 shows the proportion of individuals that were infected based on their snout-vent length (divided into five size classes). Of the seven smallest iguanas (SVL < 20 cm), six were infected with *Hepatozoon* (86%), and the infection rates declined with increasing size. Of the eight largest individuals examined (SVL > 30 cm), only one (13%) was infected. Furthermore, there was a similar relationship between body size and *Hepatozoon* infection severity; using the 16 infected individuals only, there was a significant negative correlation between number of parasites (per 50 fields-of-view, log-transformed) and SVL (*r* = −0.87, *P* < 0.0001; Figure 4). Due to the low infection prevalence, we did not compare *Plasmodium* infections with host body size.

### 3.3. Results from Molecular Analyses

**Figure 5:**
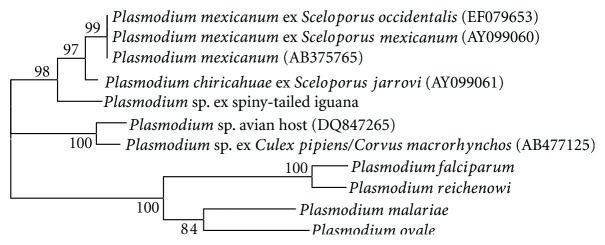
Phylogenetic relationship of the putative novel species of *Plasmodium* from *C. melanosterna* with related *Plasmodium* species.

Analysis of partial sequence (454 bp) of the cytochrome b (cytb) gene indicated that the iguana *Plasmodium* sp. was most closely related to *P. chiricahuae* (96.5% similarity) followed by *P. mexicanum* (95.8% similarity; Figure 5). Similarly, based on phylogenetic analysis, the iguana *Plasmodium *sp. was included in a clade with *P. chiricahuae *and *P. mexicanum*, both reported from *Sceloporus *spp. lizards). The cytb sequence from the iguana *Plasmodium* species has been submitted to GenBank under accession number JX849146. Efforts to identify the *Hepatozoon* species using similar molecular procedures were unsuccessful; either no amplification was noted or when bands were sequenced, no useable data was obtained, likely due to the coamplification of host and parasite DNA.

## 4. Discussion

 For parasites that are associated with declining host populations, the window of opportunity to learn about their biology is closing, and at a rate equal to the rate of host decline. Because of their small geographic range, habitat loss, and anthropogenic stressors, the black-chested spiny-tailed iguana is listed as critically endangered (one step from the “extinct in the wild” category) on the IUCN Red List [7]. In this study we identified two types of blood parasites, which likely represent novel species. To date, only two *Plasmodium* species have been reported from *Ctenosaura* species. The first is *P. rhadinurum*, which was first described by Thompson and Huff [17] in *Iguana iguana * from Mexico. Garnham [18] later observed this species in *C. similis* in Belize. We note that the morphology of gametocytes of *P. rhadinurum* (described by Telford [11]) is considerably different than the *Plasmodium* sp. we observed in *C. melanosterna*. Secondly, Mahrt [19] reported an unknown species of *Plasmodium* in *C. hemilopha *from Isla San Pedro Nolasco (an island in the Gulf of California), which he believed to be *P. mexicanum*. While our molecular analysis of the *Plasmodium* parasite indicated that it is distinct from other species with sequence data available (including *P. mexicanum*, Figure 5), we point out that there is a paucity of genetic data available for *Plasmodium* from related reptiles. Both *Sceloporus* and *Ctenosaura* are in the family Iguanidae but no comparable sequence data are available for *Plasmodium *species from any *Ctenosaura*, or any member of the subfamily Iguaninae. Additional data is needed to determine if the *Plasmodium* from *C. melanosterna* is indeed distinct or if it simply represents a morphologically variable form of *P. rhadinurum*. 

 There are only three reports of *Hepatozoon* from *Ctenosaura*, with a total of five species of *Hepatozoon* in three host species [19–21]. The morphology of our *Hepatozoon* sp. differed from all cases based on the size of gamonts and/or effects on the host cell. In a survey of 15 *C. similis* from Costa Rica [20], a novel species of *Hepatozoon* (*H. gamezi*) was described and was found in all animals (compared to 32% in the current study). The *Hepatozoon* we observed differs from *H. gamezi* in a number of ways. First, Desser [20] pointed out that the cytoplasm of erythrocytes with mature gamonts was lucent in color or became spindle shaped, while we did not observe either of these characteristics. The size of *H. gamezi* was also almost twice the size of the organism we observed (15.3 × 3.6 *μ*m versus 8.6 × 2.2 *μ*m), even though both reptile species appear to have similar erythrocyte sizes (based on figures presented in Desser [20]). Further, Desser also pointed out how the parasite of *C. similis* tended to displace the host cell nucleus, while we rarely observed this, or, if so, it was a minor deflection unless more than one parasite was present (Figure 2(c)). Finally, three *Hepatozoon* species reported from *C. pectinata* and *C. hemilopha* from Mexico also had gamonts that differed in size compared to the *Hepatozoon* we observed [19, 21].

The relationship between host body size and *Hepatozoon* infection was surprising for more than one reason. First, the relationship was evident with only 16 infected individuals (Figures 3 and 4) with smaller (younger) iguanas being more likely to be infected (Figure 3), and their parasitemias were higher (Figure 4) compared to the larger (older) iguanas. This is similar to what is seen with malaria infections in humans and human models and indicates age-related improvement of host resistance (e.g., [22–24]). Second, this pattern is opposite to that seen in hemoparasite surveys of other lizard species. For example, in *Sceloporus occidentalis*, prevalence of *P. mexicanum* increased with host body size [25], which was interpreted as older animals having a greater cumulative chance of becoming infected. Similarly, prevalence and severity of *Hepatozoon hinuliae* increased with host age/body size in the Australian lizard *Eulamprus quoyii* [26]. Furthermore, a literature review on the subject of age-related effects on blood parasites in lizards (including genus *Lacerta*, *Sceloporus*, and *Tiliqua*) indicated that higher prevalence in adults is the dominant pattern [26]. A recent exception to this was a study of parasites in tuatara (*Sphenodon punctatus*), which, like our investigation, found infections with *Hepatozoon tuatarae* decreased in frequency and severity with host size [27]. The reasons for these variable findings are unknown but could be related to the biology of the hosts, including overall size and longevity. While *C. melanosterna* and *S. punctatus* are not closely related, they are both larger than many previously examined lizard species [26]. Tuatara are long lived, able to survive over 60 years in the wild [28], and estimates from other iguanid species indicate that lifespans over 20 years are possible [29]. Determining which of these factors, if any, contributes to the divergent patterns regarding infection, and body size will require further study.

 Given the dire conservation status of this reptile, any potential source of mortality or stress should be taken into consideration. Since our study was only a cross-section of the population at one short time interval, we cannot know the long-term effects of these parasites on the animals. None of the infected iguanas were emaciated or had outward signs of disease at the time of trapping, except for occasional injuries from fighting which was frequently observed on the island (LR, unpublished observations). Results from other studies indicate that infections with *Hepatozoon* and *Plasmodium* are rarely fatal in natural reptile hosts, but they can lead to a number of sublethal effects including stunted growth, slower tail regeneration, reduced nutritional condition, lower reproductive output, and reduced competitive ability for mates (e.g., [30–32]). It stands to reason that the *Plasmodium* and *Hepatozoon* parasites of *C. melanosterna* could have similar sublethal effects over time. Despite this possibility, one could argue that if protection of biodiversity is a priority in conservation of this and other tropical species, we must strive to ensure the survival of both the host and its parasites, especially if the parasites are destined to be lost if the host goes extinct.
